# Modern Sociology

**Published:** 1899-04-08

**Authors:** 


### Modern Sociology.

### MEN AND MACHINERY.

From a Correspondent.

It is a favourite complaint among a certain class of people, with higher pretensions to æsthetic culture than to economic knowledge, that the advent of machinery took away from the craftsman—it is a characteristic of these good people that they always say "craftsman" for "workman"—the joy in his work that he had in mediæval times. Thereby, they say, we have injured the artistic value of his work, and such modern advantages as accuracy, neatness, and the like do not count for anything in their eyes. As for the fact that a machine can turn out a score of identical articles in the time it took the old craftsman to produce one, that is only an added offence in their eyes, and one would think from their talk that the end of all gladness and well-being in the world has come because you can order a gross of porridge-bowls all of the same pattern. Now it may be doubted if the craftsman of the Middle Ages took quite as much joy in his work as these amiable gentlemen and ladies think. The artist did, and there is no reason on earth why the artist should not do so now. But the average workman is not artistic, and does not do any better for being left to his own devices in the matter of taste. Moreover, there are many things in which art is of no particular service.

Even in things which are in themselves artistic, there are many details of making which have little to do with art. There is a pretty story told about a workman who at the dedication of Cologne Cathedral, exclaimed, "How little I knew that I was making *this*." He had been employed for a great part of a lifetime in mixing mortar for the masons, and only at the last had it dawned on him how beautiful was the fabric he had helped to bind together. Now the only difference between this man and other mortar-mixers was that he had lived to see the cathedral finished, and that he had the eyes to appreciate its beauty. Many of his fellow-workmen had died before the great work was completed, and more, having received their wages, cared only to know if they were likely to get another job. And, at the best, the mixing of mortar is not an artistic piece of work, though if it be not well done, the finest fabric will be the sooner lost to the world. But if a machine could do the work as well as a man, there would be little cause for regret.

Let us not be carried away by the preservation of some exquisite piece of old work into a hysteric and foolish lamentation over the days that are no more. That piece was preserved probably because it was artistic, and if we could go back into a mediæval house, we should find half its furnishings as commonplace as our own. On the other hand, let us consider what machinery has done for the workman. We do not mean only in the matter of increased production, whereby the poorest among us has more comforts of every kind than in former times even the rich could secure. That the progress of invention has added to the comfort of the consumer in every rank is such an obvious thing that no one ventures to deny it. But not less has the development of machinery benefited the workman, as workman. Terrible as it may seem to the æsthetics, all good work tends to become more and more automatic. The best workman is the one who can produce a hundred things of the same kind with as little difference between them as possible. This implies a specially-trained dexterity, which in turn implies the application of much attention and intelligence. The intelligence in time becomes quasi-automatic, but never so much so as a perfect machine which does the same kind of work. Therefore, a man if he spends his day in feeding a machine with the raw material for a certain article, instead of making the article himself, will at the end of the day be much less fatigued than if he had had to bring to bear the attention, intelligence, and manual work required under the old system to do the work himself. Professor Marshall, in his "Economics of Industry," instances the jack-plane, as an instance of the benefit machinery confers on the workman. The jack-plane is the tool used to smooth boards for floors and the like. The use of it was fatiguing, but mechanical, and developed no high order of intelligence. All the work it used to do is now done by a machine. Who suffers by this? The work is more perfectly done, so the employer and the consumer have no reason to complain. But has the workman? Is it not rather the case that, this mechanical work being done for him, save for the matter of supervision, he can give more attention to those more interesting and artistic parts of the work, which under the old *régime* were the prerogative of a favoured few. But, it may be objected, the work does not afford opportunities even now for artistic work on the part of all. But even suppose that our ex-jack-planer has no outlet in his daily work for his intelligence or his artistic capacities, does it count for nothing that at the end of the day he is not so exhausted by the mere muscular effort of his day's work as to be unable to profit by the opportunities for culture that are commoner now than ever before? In the matter of health, too, he is the gainer. Adam Smith pointed out in his great work that every industry developed weaknesses of its own. This was true in his day, and in the early days of the factory system there is no doubt that health was often sacrificed to production. But this is becoming less and less the case every year. The majority of workshops are more sanitary than workshops ever were before, and, with little but the task of supervision left to the workman, he is not so likely to overstrain himself in order to accomplish a little more work in the day as used to be the case. Smith said that a carpenter was not supposed to last in his prime for more than eight years. What is the average now? Even coal-mining is no longer the unhealthy occupation it used to be, and the same is true of almost every branch of industry.

Moreover, there is the advantage of greater adaptability. Machines so similar in construction and use are employed in trades which seem quite diverse, that a man possessed of general ability can move with far more ease from one trade to another than he could formerly do. The more mechanical a man's work is the more easily can he transfer his work from one branch of industry to another. In short, the workman of to-day gains more and suffers less from his work than did his predecessor of any previous age. And if he is not an artist, the fault is his own, for the means of study lie more ready to his hand than ever before. If he has no artistic sense now, no joy in his work, he probably would never have had any, even though he had lived in the heart of the Middle Ages.

